# The Impact of Structural Variations and Coating Techniques on the Microwave Properties of Woven Fabrics Coated with PEDOT:PSS Composition

**DOI:** 10.3390/polym15214224

**Published:** 2023-10-25

**Authors:** Vitalija Rubežienė, Sandra Varnaitė-Žuravliova, Audronė Sankauskaitė, Julija Pupeikė, Paulius Ragulis, Aušra Abraitienė

**Affiliations:** 1 Department of Textile Technologies, Center for Physical Sciences and Technology, 48485 Kaunas, Lithuania; vitalija.rubeziene@ftmc.lt (V.R.); audrone.sankauskaite@ftmc.lt (A.S.); julija.pupeike@ftmc.lt (J.P.);; 2 Physical Technology Department, Center for Physical Sciences and Technology, 10257 Vilnius, Lithuania; paulius.ragulis@ftmc.lt

**Keywords:** conductive textiles, microwave absorbing materials, electromagnetic radiation

## Abstract

Minimizing the impact of electromagnetic radiation (EMR) holds paramount importance in safeguarding individuals who frequently utilize electrical and electronic devices. Electrically conductive textiles, which possess specialized EMR shielding features, present a promising solution to mitigate the risks related to EMR. Furthermore, these textile-based shielding materials could find application as radar-absorbing materials in stealth technology, emphasizing the need for substantial absorption capabilities in shielding mechanisms. In this study, various textile-based materials with an electrically conductive coating that contain the conjugated polymer system poly(3,4-ethylene-dioxythiophene)-polystyrene sulfonate (PEDOT:PSS) were prepared and investigated. The influence of the textile substrate structural parameters, coating deposit, and coating method on their microwave properties—transmission, reflection, and absorption—was investigated. Reflection and transmission measurements were conducted within a frequency range of 2 to 18 GHz. These measurements revealed that, for the tested samples, the shielding properties are determined by the combined effect of reflection and absorption. However, the role of these two parameters varies across the tested frequency range. It was defined that for fabrics coated on one side, better reflection reduction is obtained when the shielding effectiveness (SE) is below |20| dB. It was found that by controlling the coating deposition on the fabric, it is possible to fine-tune the electrical properties to a certain extent, thereby influencing the microwave properties of the coated fabrics. The studies of prepared samples have shown that reflection and transmission parameters depend not only on the type and quantity of conductive paste applied to the fabric but also on the fabric’s construction parameters and the coating technique used. It was found that the denser the substrate used for coating, the more conductive paste solidifies on the surface, forming a thicker coat on the top. For conductive fabrics with the same substrate to achieve a particular SE value using the knife-over-roll coating technology, the required coating deposit amount is considerably lower as compared with the deposit necessary in the case of screen printing: for the knife-over-roll-coated sample to reach SE 15 dB, the required deposit is approximately 14 g/m^2^; meanwhile, for a sample coated via screen printing, this amount rises to 23 g/m^2^.

## 1. Introduction

Protection against radio frequency (RF) electromagnetic fields has become important due to the increasing number of electronic and telecommunication devices in commercial, military, and scientific use [1,2,3,4,5]. RF radiation pollution is the fourth major concern, following air pollution, water pollution, and noise pollution [6].

Within the RF frequency range, the microwave range stands as the most utilized segment, defined within the range of 1–40 GHz. This range is predominantly favored for contemporary wireless, point-to-point, and satellite communications.

RF radiation falls into the category of non-ionizing radiation. However, although RF radiation is not as hazardous as ionizing radiation, RF electromagnetic fields can have a negative impact on people who frequently use devices generating such electromagnetic pollution. In 2011, a Working Group of the World Health Organization’s International Agency for Research on Cancer (IARC) classified RF radiation as a possible human carcinogen (Group 2B) [7]. Moreover, the recent epidemiological studies strengthen and support the conclusion that RF radiation should be categorized as carcinogenic to humans (IARC Group 1) [8].

Furthermore, this electromagnetic energy poses a risk not only to operators working with specific devices but also to other electrical equipment. It can generate electromagnetic interference (EMI), which has the potential to disrupt the performance of other equipment, leading to undesirable responses or operational failures [1,9,10].

Electrically conductive textile-based materials that possess specific electromagnetic radiation shielding properties provide an opportunity to mitigate the aforementioned threats. Fabrics that incorporate conductive additives (e.g., metalized yarns) or are coated with conductive formulations interact with the electrical field components of electromagnetic waves.

The extent of EMR reduction significantly depends on several key factors: the choice of material, material thickness, and shield thickness; and the frequency of the fields under consideration [11,12]. The attenuation of EMR by the designated shielding material critically depends on its conductivity and electromagnetic properties, such as complex permittivity and complex permeability [12].

The desired property of EMR-shielding textile materials is low transmission, which means high shielding effectiveness (SE, dB). The desired characteristic of shielding materials is low transmission, denoting high shielding effectiveness (SE) measured in decibels (dB), which can be achieved through reflection, absorption, or a combination of both. In some cases, the use of EMR reflection as the main component in shielding could be unfavorable, as it can be the cause of disturbances in the operation of other electronic equipment, as well as a bigger hazard to operators [10,13,14].

Considering the secondary electromagnetic irradiation through reflection by conductive materials, absorption is frequently regarded as the preferred mechanism for electromagnetic radiation (EMR) shielding [15]. This type of EMR shielding is gaining more significance due to the growing proliferation of emitters and specific electrical equipment.

Additionally, when EMR shielding fabric is applicable as radar-absorbing material in stealth technology, a substantial contribution to shielding should come from absorption [4,5,16].

Hence, both the reflection and absorption properties are crucial in the study of shielding materials, as the impact of each feature depends on the intended application of the material.

A wide variety of metallized textile-based shielding materials, which are known for their high reflection factors, are readily available in the market [10,17]. However, these materials often lack the ability to absorb EMR, a critical feature in numerous scenarios, such as shields for electronic equipment located in areas with continuous human occupancy or in the construction of anti-radar shields [18,19,20].

In recent years, transition metal carbides, nitrides, and carbonitrides (MXenes) have garnered the attention of researchers for the development of MXene-based EMI shielding materials [21]. Recent research on MXene-based textiles has demonstrated that materials of this kind exhibit significant potential in shielding applications due to their conductivity, flexibility, and ease of coating [22,23]. However, the synthesis of MXenes remains challenging due to their instability after exfoliation [24].

Another interesting novel textile-based EMR shielding material was developed by Liang et al. [25]. They prepared a solid–solid phase change material (PCM) coating which was used to encapsulate polymer textiles decorated with silver nanowires (AgNWs) based on a scalable dip-coating strategy. They obtained phase change textiles with a shielding effectiveness of approximately 72 dB at a thickness of 0.26 mm. However, in this case, shielding was mainly achieved through reflectance.

Designing fabrics that are capable of effectively absorbing electromagnetic radiation is considerably more challenging than designing textile materials that act as reflective shields. For EMR-absorbing textile materials, the attainable level of barrier properties depends on the structure of the base fabric (substrate) and the nature and thickness of the shield, and these factors are also variable as a function of EMR frequency [26,27,28].

Conversely to commonly used metallic shielding materials, intrinsically conducting polymers (ICPs) possess the capability to both reflect and absorb electromagnetic radiation (EMR) in the microwave frequency range [19,26]. This dual shielding characteristic, which includes absorption in addition to reflection, distinguishes ICPs from metals and makes them more promising materials for applications that require high EMR shielding effectiveness, as well as absorption dominant shielding, for instance, such as in the realm of military stealth technology.

Previous research related to EMR shielding using intrinsically conducting polymers on textile fabrics has primarily focused on the applications of polyaniline (PANI) and polypyrrole (PPY) [7,18]. However, there is an increasing trend in publications focusing on the application of other ICPs, e.g., poly(3,4-ethylenedioxythiophene (PEDOT), for the development of EMR shielding textiles [26,29]. In textile applications, the conjugated polymer system-poly(3,4-ethylenedioxythiophene)–polystyrene sulfonate (PEDOT:PSS) is predominantly used. Here, PEDOT is combined with a water-soluble polymer, poly(styrene sulfonic acid (PSS), to enhance its solubility in water. PEDOT:PSS has comparatively high conductivity, good thermal and hydrolytic stability, and favorable processability in aqueous dispersions [30,31,32,33].

Furthermore, PEDOT:PSS offers additional advantages. For instance, hydrogel particles provide exceptional processing characteristics for creating thin, transparent conducting films [26], and coating with compositions containing PEDOT:PSS has no adverse impact on the mechanical properties of the substrate, enabling its use as a flexible material [34].

Various formulations containing PEDOT:PSS are currently commercially available not only for applications such as sensors, thermoelectric devices, and solar cells but also for textile-based functional materials or smart textile.

Most often, compositions with PEDOT:PSS are used for the development of electronic textiles, which are classified as smart textiles [31,32,35]. Such materials are functional textile materials, which interact actively with their environment, i.e., by responding or adapting to changes in the environment [36].

Numerous researchers have worked on preparing conductive fabrics treated with formulations containing PEDOT:PSS for applications such as sensors [37,38], data storage and transmission [39], and heating textiles [40,41,42].

However, there are fewer studies in which fabrics with compositions containing PEDOT:PSS are utilized for EMR shielding.

Ghosh et al. [30] manufactured a cotton fabric based on PEG/PEDOT:PSS by using the dip-coating method. Their findings demonstrated that the modified cotton fabric achieved a notable electromagnetic interference (EMI) shielding effectiveness (SE) of approximately 46.8 dB in the X-band frequency range, coupled with an electrical conductivity of about 51 S/cm after 20 dipping cycles. The enhancement in conductivity was obtained due to the use of polyethylene glycol (PEG), which acted as an adhesion promoter, as well as a booster for phase segregation agent. Also, the sum of absorption (SE_A_) and the sum of reflection effectiveness (SE_R_) from the total SE were separately calculated. The obtained values of SE_A_ and SE_R_ indicated that the absorption-dominant shielding mechanism was at play in the coated cotton fabrics. It’s worth noting that after 20 dipping cycles, the thickness of the coated fabric reached approximately 0.38 mm, whereas the apparent thickness of the untreated cotton fabric was roughly 0.18 mm. This indicates that the material’s thickness increased by more than two-fold after the coating process. Furthermore, it requires as many as five dipping cycles to achieve the necessary shielding effectiveness of 20 dB, which is crucial for commercial applications. This technology appears to have limited scalability.

Islam et al. [43] developed a reduced graphene oxide/PEDOT:PSS-based knitted cotton fabric for EMI shielding applications. The modified cotton fabric exhibited outstanding electromagnetic interference shielding effectiveness, reaching up to 49 dB in the frequency range of 30–1530 MHz and 39 dB in the X band. However, in this case, the reflection dominated.

Sharma and James [44] used the conductive polymer PEDOT:PSS in a composition with polyvinylpyrrolidone (PVP) to coat carbonized electrospun polyacrylonitrile (PAN) nanofibers, with PVP serving as a binder and booster for the phase segregation of conducting PEDOT from insulating PSS. It was reported that the prepared composites exhibited EMI shielding through an absorption-dominated mechanism rather than reflection. The proposed method is exceptionally suitable only for the fabrication of nanofibers with shielding properties.

The solubility of PEDOT:PSS in water and the loss of conductivity, which determines the shielding effectiveness, after washing procedures are the primary drawbacks of these composites [40]. To enhance these properties, improvements can be made by altering the synthesis method of PEDOT:PSS, incorporating nanoparticles or combining different polymers [33,40,45], or by preparing the fabric or fiber surface through chemical or thermal pretreatment to achieve adhesion on textile surfaces [26,40,46].

Despite the significant amount of completed research on PEDOT:PSS-based EMR shielding textiles, fabrics that combine high electrical conductivity and exceptional wear resistance and robust mechanical stability have not yet been successfully developed.

The simplest processes for applying compositions containing PEDOT:PSS to textile fabrics include screen printing, inkjet printing, knife-over-roll technology, or immersing (solution coating). Usually, when the coating is applied as a very thin layer on the fabric structures, it does not significantly alter the flexibility of the fabrics [5,26,33].

The electrical characteristics of coated fabrics are influenced by various factors, including the concentration of reactants, the quantity and thickness of the polymer coating, the nature of the substrate surface, the type of fibers used, the adhesive strength of the coating to the textile surface, and more [17,26,46]. Substrate pretreatment before coating is relevant, as chemical and physical surface properties play an important role in absorption reactions [46,47].

Structural parameters of the substrate fabric, such as thickness, porosity, and mass per unit area, also affect the overall electromagnetic shielding effectiveness of coated conductive fabric, as coating composition diffuses and penetrates differently into various structures. However, there is a lack of publications on this topic.

Therefore, further research is necessary to collect sufficient data for predicting the behavior of conductive fabrics based on PEDOT:PSS and the integration of this type of polymer into EMR shielding textiles.

The aim of this work was to develop and investigate various textile-based materials with an electrically conductive coating containing PEDOT:PSS for EMR shielding applications. The influences of the textile substrate structural parameters, coating deposit, and coating method on their microwave properties—transmission, reflection, and absorption—were investigated in this research. For this purpose, various textile substrates were coated using thin-layer coating techniques, enabling the development of innovative lightweight, thin textile protective shields and specialized protective clothing, including radar-absorbing materials for soldiers.

The developed fabrics are intended for EMR shielding in the microwave range, as well as for absorbing microwaves in the radar operating range to function as radar-absorbing materials (RAMs). Consequently, the measurements of the reflection and transmission of the developed textile fabrics were performed within a frequency range of 2–18 GHz, covering the defined frequencies relevant to the application.

The generated conductive fabrics are designed to function as shields against electromagnetic radiation in the microwave range, with pronounced absorption capabilities within the radar operating range, thus serving as radar-absorbing materials (RAMs). Accordingly, measurements of the reflection and transmission properties of these textiles were conducted over a frequency range of 2–18 GHz, covering the specific frequencies relevant to these applications.

## 2. Materials and Methods

### 2.1. Materials

**Figure 1:**
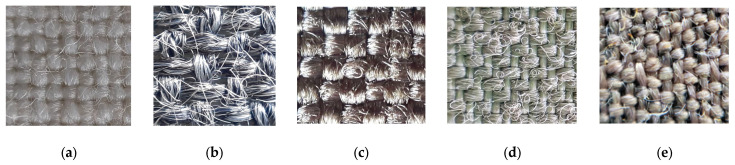
Views of substrates (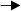
—weft direction; 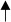
—warp direction): (**a**) PC, (**b**) NV, (**c**) C, (**d**) P, and (**e**) AV.

Five groups of coated samples, consisting of various woven fabrics used as substrates, were prepared for investigation. The details of the substrates used for coating are presented in Table 1. The images of substrates, taken with a phonoscope (Leuchtturm, Geesthacht, Germany) mounted on the phone, are presented in Figure 1. The magnification of the phonescope glass lens was 60×.

The conductive paste Clevios SV3 (see Table 2), manufactured by Heraeus (Hanau, Germany), was used to coat the substrates described in Table 1 and shown in Figure 1.

#### Coating of Substrates with Conductive Paste

In order to prepare the samples for investigations, substrates of various woven fabrics (see Table 1 and Figure 1) were coated with a conductive paste containing PEDOT:PSS (see Table 2), using two different conventional textile coating technologies: screen-printing and knife-over-roll coating.

For the screen printing of samples, a laboratory screen-printing set was utilized. After the screen printing, the samples underwent a drying process in a laboratory oven and steamer TFOS IM 350 (Roaches International, Birstall, UK), where they were exposed to a condensation temperature of 150 °C for 4 min. The main objective of this step was to enhance the adhesion and bonding of the coating to the substrate. The thickness of the coating and its deposit were adjusted by varying the number of passes.

A pilot continuous coating and laminating machine called “Rotolabo Multi 600” (Matex, Brendola, Italy) was employed for the coating of textiles using the knife-over-roll technique. The knife was positioned over the textile fabric with a roll during a knife-over-roll coating. The gap between the knife and the fabric varied from 0.1 mm to 0.5 mm to control the coating thickness. For the thermofixation of the prepared samples, the same temperature (150 °C) and duration (4 min) as after screen printing were applied.

### 2.2. Methods

#### 2.2.1. Determination of Structural Parameters

Mean values from five specimens were used to calculate the mass per unit area, following the standard test method EN 12127 [48]. The standard deviation of the results for all tested fabrics was less than 1 g/m^2^.

Five specimens from each fabric were employed to calculate the mean thread count in square centimeters following the standard test method EN 1049-2 [49]. The standard deviation of the results for all tested fabrics was about 0.5 in cm^2^.

The Schmidt control instrument with a resolution of 0.01 mm was utilized to measure the sample thickness, in accordance with EN ISO 5084 standard [50]. The mean values from five measurements are presented in Table 1. The standard deviation of the results for all tested fabrics was less than 0.5 mm.

To assess the porosity, the air permeability of the fabrics was measured in accordance with the EN ISO 9237 [51] standard. The Frazier 2000^TM^ (Frazier Precision Instr Co., Hagerstown, MD, USA) differential pressure air permeability tester FAP-1034-LP was employed, applying a pressure drop of 100 Pa across a test surface area of 20 cm^2^. The air passed through the face side of the fabrics. The average values from 10 measurements for each sample are presented as a result in Table 1. The standard deviation for all tested fabrics was within a 5 mm/s range.

The coating deposit amount (g/m^2^) on the coated samples was estimated by calculating the difference between the mass per unite area of produced coated fabrics and that of the substrate fabric.

The conditioning and determination of samples’ structural parameters were conducted in a standard atmosphere, with a temperature of (20 ± 2) °C and relative humidity of (64 ± 4)%, in accordance with the EN ISO 139 [52] standard.

#### 2.2.2. Moisture Management

A moisture management tester (MMT) was utilized to determine the multidirectional liquid moisture transport capabilities of the tested samples. The investigations were carried out according to the AATCC Test Method 195 [53]. Five specimens, each with dimensions of (8 × 8) cm, were prepared for each type of sample. The conditioning of samples and testing took place in a standard atmosphere: temperature of (20 ± 2) °C and relative humidity of (64 ± 4)%, as per the EN ISO 139 [52] standard.

Only two parameters were considered during this investigation: the wetting time and absorption rate. These parameters are more critical for assessing the penetration of the conductive paste into the material than other moisture management properties determined by this method.

The wetting time is the duration it takes for both the top and bottom surfaces of the specimen to become wet after the test commences.

The absorption rate is the average speed of liquid moisture absorption for the top-to-bottom surfaces of the specimen during the initial change of water content during the test.

The results of mean parameters tested for substrates are presented in Table 1.

According to AATCC Test Method 195 [53], the indices are graded and converted from values to grades based on a five-grade scale. Table 3 shows the range of values converted into grades.

#### 2.2.3. Aqueous Liquid Repellency Test

To assess the surface properties of substrates before coating, the fabrics were subjected to an aqueous liquid repellency assessment, following the ISO 23232 standard [54], commonly referred to as the “drop test”.

The aqueous solution repellency grade of a substrate represents the numerical value of the highest index that does not cause the substrate to become wet within a specific time frame of (10 ± 2) s. A grade of zero (0) is assigned when the substrate does not pass the test with the 98% water solution. Three different places are tested to provide a mean aqueous solution repellency grade for the sample.

The higher the aqueous solution repellency grade, the better the resistance to staining by liquid aqueous substances, thus indicating higher hydrophobicity. Conversely, the lower the grade, the greater the hydrophilicity of the fabric. The results of aqueous solution repellency for substrates are presented in Table 1.

#### 2.2.4. SEM Analysis

Scanning electron microscopy (SEM) was used to examine the surface morphology of the coated samples with a Quanta 200 FEG device (FEI, Eindhoven, The Netherlands) at 20 keV (low vacuum). The technical and technological parameters used were as follows: electron beam heating voltage—20.00 kV; beam spot—5.0; work distance—6.00 mm; low vacuum—80 Pa; detector—LFD; and magnification at 5000×, 10,000×, and 20,000×.

#### 2.2.5. Evaluation of Electrical Conductivity

The electrical properties of the coated samples produced for this research work were characterized using the four-electrode, four-wire KELVIN method according to EN 16812 [55] standard.

The width of the specimen (d, cm) 2 cm, with a distance between measuring electrodes 10 cm. The current (I, A) of 0.001 A was applied during the test. The voltage (U, V) for each specimen (five specimens for each sample were involved in the measurements) was measured every 10 s for one minute, and a mean value was used for further calculations. The conditioning of samples and testing were performed under a standard atmosphere: temperature of (20 ± 2) °C and relative humidity of (64 ± 4)%, according to EN ISO 139 [52].

Both the resistance (R, Ω) and linear resistance (R_L,_ Ω/cm) were calculated according to Ohm’s law, using Equations (1) and (2). The calculated average value of *R_L_* was presented as a result, with a standard deviation of less than 2.0 Ω/cm.

(1) $$R=\frac{U}{I}$$

(2) $$R_{L}=\frac{R}{d}$$

#### 2.2.6. Microwave Measurements

**Figure 2:**
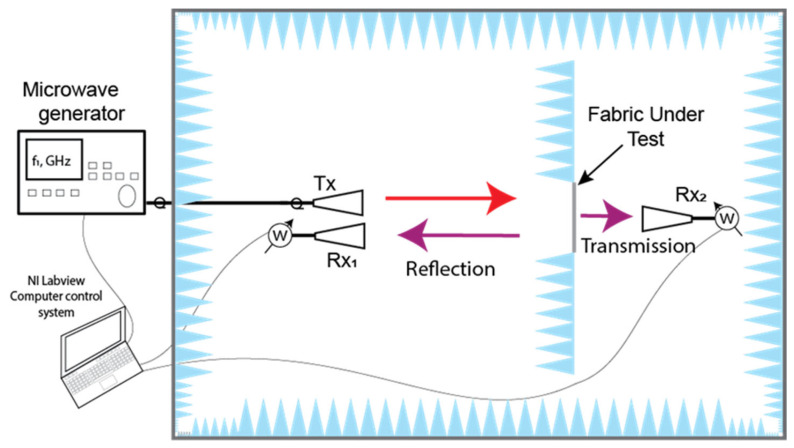
Measurement setup of microwave transmission and reflection from the coated fabric. Tx indicates transmitting antennas, and Rx_1,2_ indicates receiving antennas.

The examination of the reflective (Γ) and transmissive (T) characteristics of the newly designed textile fabrics was conducted across a frequency spectrum spanning from 2 to 18 GHz, encompassing the S, C, X, and Ku bands (2–4 GHz, 4–8 GHz, 8–12 GHz, and 12–18 GHz, respectively). These tests took place within a semi-anechoic chamber, and the illustration of the measurement setup can be found in Figure 2.

In the semi-anechoic chamber, the sampled material (sized at 20 × 20 cm) was enclosed by an absorber sheet to obstruct diffracted waves from directly reaching the receiving antenna. A tunable microwave generator was used as a microwave source. A continuous wave signal was directed to transmitting antenna Tx during the measurement. A series of horn antennas was employed for measurements within different frequency ranges: 2.4–4.0 GHz (S band WR284), 4–7.5 GHz (C band WR159), 7.5–12.4 GHz (X band WR90), and 12.4–18 GHz (Ku band WR62). These antennas were utilized in conjunction with the Rohde & Schwarz average power sensor NRP-Z24 from Rhode & Schwarz (Munich, Germany) that was connected to the receiving antennas through the respective waveguide-to-coaxial line adaptors. These sensors boasted a maximum absolute uncertainty of 0.222 dB, equivalent to ±5.24%. The measuring setup enabled the assessment of electromagnetic radiation (EMR) shielding effectiveness up to a maximum of 35 dB. The expanded uncertainty for both the shielding effectiveness and reflection measurements is within ±7.4% for a single data point at a fixed frequency. This reported expanded uncertainty is derived from a standard uncertainty value multiplied by a coverage factor (k = 2), providing a confidence level of approximately 95%.

For the reflection measurement, the receiving antenna Rx_1_ was positioned in proximity to the transmitting antenna Tx, with a direct coupling lower than –30 dB. The transmitted power, P_t_ (W), with the sample under testing, was normalized against the power P_t0_ (W) measured in its absence. Therefore, the transmittance can be expressed as follows:

(3) $$T=\frac{P_{t}}{P_{t0}}$$

The results of the transmittance (T) are presented as the EMR shielding effectiveness (SE, dB), expressed in decibels:

(4) SE=10×logT

To determine the microwave reflectance of the tested textile samples, the reflected microwave power from the fabric, denoted as P_r_, is divided by the reflected power, P_m_, which is measured when the sample under test is substituted with a stainless-steel plate of identical dimensions. This calculation allows us to obtain the reflectance value (Γ):

(5) $$\Gamma =\frac{P_{r}}{P_{m}}$$

The absorption (A) in a tested sample was determined as the difference among falling, transmitted (T), and reflected (Γ) waves:

(6) A=1−T−Γ

## 3. Results

To develop materials designed for EMR shielding and absorption in the radar operating range, woven fabrics with different structures and various fiber compositions were coated with a formulation containing PEDOT:PSS. Our primary focus during this investigation was centered on the microwave properties of these materials.

The coating techniques, namely knife-over-roll and screen printing, were applied in the research with the aim of creating fabrics with a conductive layer on the backside only, while providing unique functionalities on the face side. For instance, this could entail camouflage pattern printing or other specialized features for protective clothing.

### 3.1. Correlation between Electrical Conductivity and Shielding Effectiveness

To absorb or reflect EMR, materials must interact with the electric or magnetic field of the radiation. Textiles are not inherently EMR shielding materials. However, after the incorporation of electrically conductive additives, they can interact with the electric component of EMR [4,14,26].

The EMR shielding efficiency of a material is characterized by its electrical conductivity, permittivity, and permeability; the parameters of the source; and the properties of the ambient surrounding [4,5,40]. The mechanism of EMR shielding for fabrics coated with a conductive coating was explained in detail in our previous study [46].

Typically, to assess the electrical conductivity of a textile material, the inverse of its resistance is measured. There are some standard test methods for the determination of the electrical resistance of the fabrics. Examples include the following: EN 1149-1 [56]—measurement of surface resistivity; EN 1149-2 [57]—measurement of the electrical resistance through a material (vertical resistance); and EN 16812 [55]—measurement of linear electrical resistance. Nonstandard methods are also widely used [19,32,35].

It has been stated [20,58] that, for fabrics with metalized yarns, the most suitable is the prediction of SE based on volume resistivity. However, another work [17] in which fabrics with different conductive additives were investigated showed that the surface resistivity has a better prediction ability compared to volume resistivity.

To determine the relationship between SE and electrical conductivity of the developed samples, the correlation between the EMR shielding efficiency (SE, dB) and electrical resistance of fabrics with various fiber compositions and different structures coated with formulations containing PEDOT:PSS was studied.

In our previous study [46], it was stated that fabrics coated with conductive paste can be considered as a thin layer exhibiting specific surface conductivity (σ = 1/Rs), where Rs represents surface resistance. Given that the fabric thickness is significantly smaller than the wavelength of the electromagnetic waves used in experiments, the dielectric properties of the fabric may be disregarded.

A recent analysis of the relationship between SE and surface resistivity for different woven fabrics coated with conductive paste containing PEDOT:PSS was given in our previous study [46]. The results obtained showed that a correlation between these parameters was very weak (the coefficient of determination was R^2^ = 0.255). It is reasonable to conclude that the results of surface resistivity measurements for samples coated with conductive paste and possessing nonuniform surfaces are not consistent and reliable. This is attributed to the method employed, which fails to eliminate contact resistance within the system.

The relationship between SE and linear resistance, which was measured according to the four-electrode, four-wire KELVIN method—EN 16812 [55]—was investigated in this study.

The measurement results of linear resistance (Ω/cm) (see Supplementary Table S1: Mean value of linear resistance of different fabrics coated with various amounts of conductive paste containing PEDOT:PSS) indicate that, for each different type of fabric, the SE increases, and the linear resistance decreases with an increase in the coating deposit.

**Figure 3:**
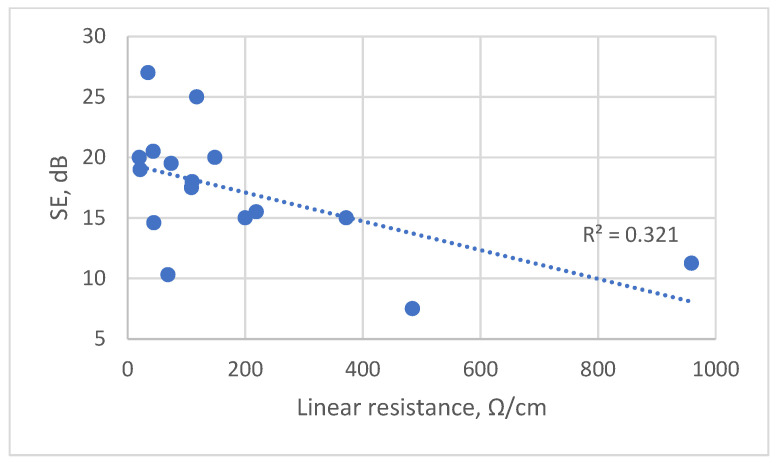
Scatter diagram with statistical analysis of linear resistance of tested samples coated with conductive paste on EMR shielding effectiveness (SE).

However, when assessing the overall dependence between SE and linear resistance in all tested fabrics, a low correlation between these parameters is observed with the coefficient of determination (R^2^), which reaches only 0.32 (see Figure 3). A similar low correlation (R^2^ = 0.18) is seen between the coating deposit and linear resistance (see Supplementary Figure S1: Dependence of linear resistance on coating deposit).

Therefore, the obtained linear resistance values do not allow us to compare the conductivity of different conductive textile structures and to objectively predict the shielding ability of such fabrics, due to the problematic contact between the coated fabric and the measurement equipment.

Thus, the most reliable method for determining the SE of conductive materials with different structures is by actually measuring it.

### 3.2. Microwave Properties of Coated Fabrics

The primary objective behind developing fabrics with conductive coatings was to endow them with the capability to shield against EMR in the microwave range and to investigate their reflectance and absorption properties within this range.

Dismounted soldiers face a significant threat from battlefield radars that typically operate in the X and Ku bands, at 8–12 GHz and 12–18 GHz, respectively [59]. To address this threat, measurements of the reflection and transmission were conducted over a frequency range of 2–18 GHz. This range encompasses the specific frequencies relevant to the intended application. The absorption in a tested samples was determined as the difference between the transmitted and reflected waves (see Formula (6)).

We did not aim at obtaining excellent SE (i.e., SE > 30 dB) during this research because it is known [19,26] that, for radar signature reduction purposes, it is essential that the SE is not excessively high, as an excessively high SE would render the textile material too reflective, leading to weak radar protection properties. In most cases of EMR shielding applications, SE of 20 dB is typically required, signifying that 99% of the electromagnetic energy is either reflected or absorbed by the material [60].

#### 3.2.1. Electromagnetic Shielding Ability of Coated Fabrics

**Figure 4:**
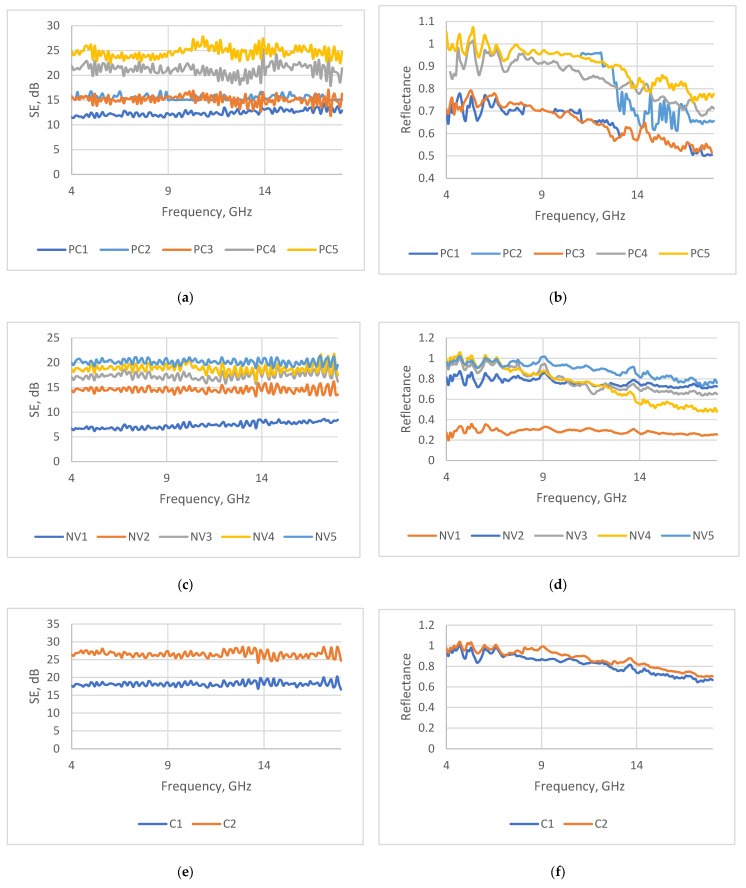

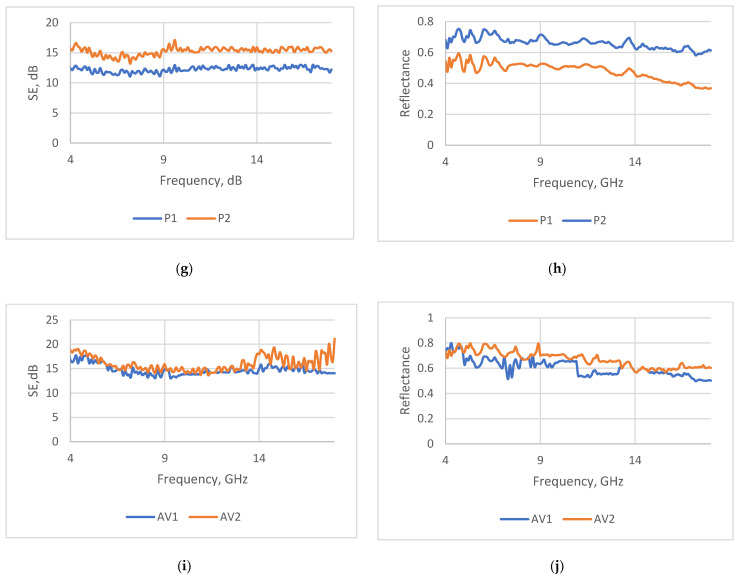
Shielding effectiveness (SE) and reflectance (R) dependence on the coating deposit for each different type of fabric coated via the knife-over-roll technique over the whole tested frequency range: (**a**) shielding of samples PC1, PC2, PC3, PC4, PC5, (**b**) reflectance of samples PC1, PC2, PC3, PC4, PC5, (**c**) shielding of samples NV1, NV2, NV3, NV4, NV5, (**d**) reflectance of samples NV1, NV2, NV3, NV4, NV5, (**e**) shielding of samples C1, C2, (**f**) reflectance of samples C1, C2, (**g**) shielding of samples P1, P2, (**h**) reflectance of samples P1, P2, (**i**) shielding of samples AV1, AV2, (**j**) reflectance of samples AV1, AV2.

The microwave properties and the values of the coating deposit of the tested samples are presented in Supplementary Tables S2 and S3. The data in Supplementary Table S2 are for samples coated using the knife-over-roll technique, and the data in Table S3 are for samples coated via screen printing. In general, the data presented in these tables reveal the contribution of reflection and absorption to the total SE for each tested sample at 10 GHz, 12 GHz, and 18 GHz, respectively. For better data visualization, the results of transmission and reflection measurements for some samples are presented graphically over the whole tested frequency range (see Figure 4).

The transmission and reflection measurements revealed that, for the tested samples, the shielding properties are predominantly influenced by the combined effect of reflection and absorption. However, the roles of these two parameters vary across the tested frequency range.

The shielding effectiveness (SE) of the investigated coated fabrics is steady in all tested frequency ranges and are mostly sufficiently good for fabrics intended for EMR shielding and radar absorbing. In the meantime, the reflection ability and, consequently, the absorption ability are frequency-dependent. A better absorption ability for all tested samples was obtained at the end of the testing frequency range, namely at 16–18 GHz.

Nonetheless, within the realm of EMR shielding textile-based materials, only those with a significant capability for absorption contribute meaningfully to their potential use as radar-absorbing materials (RAMs) [16,61]. These materials should feature the lowest reflection and transmission coefficients, as well as the greatest absorption factor [62].

During the analysis of results obtained for the different investigated fabrics coated with conductive paste containing PEDOT:PSS, the research revealed that superior absorption capabilities were achieved when the total SE value was below 20 dB. Consequently, to be effective at reducing radar signatures, SE should not be excessively high, as this would render the textile material overly reflective.

On the other hand, the SE must also not be too low, as a material with a transmission that is too high can lead to poor radar protection properties. Hence, radar-absorbing textile materials should have a shielding effectiveness of −20 dB ≥ SE > 10 dB, which is classified as a good grade, according to the requirements of EMR shielding textiles [52].

The analysis of the data presented in Table S2 (data of samples coated using the knife-over-roll technique) and Table S3 (data of samples coated using the screen-printing technique) indicates that textile fabrics coated with a composition containing the conductive polymer PEDOT:PSS demonstrated certain microwave absorption properties. However, only some samples demonstrated a rather tolerable absorption ability (absorption ≥ 40%), along with sufficiently good shielding effectiveness (SE > 12 dB); these are samples PC1, PC3, P1, AV1, and SPC2.

The obtained relatively low absorption ability can be explained by the fact that the absorption is a bulk-related (or thickness-related) phenomenon [12,61]. The developed samples have a very thin coating layer, meaning that the shield thickness of such coated materials is not sufficient enough to achieve the required contribution of absorption to the total EMR shielding. Multilayer shielding textile systems are more promising to achieve a higher absorption coefficient [10,12,63]. Therefore, materials consisting of several layers were developed, where these layers contribute to a gradual attenuation of the incident wave. Research on such materials will be presented in the next study.

#### 3.2.2. Influence of Coating Deposit and Textile Substrate Structure on Shielding Properties

The EMR shielding effectiveness of coated fabrics strongly depends on the formation of a three-dimensional conductive coating network, which settles on the surface of the fabric and penetrates deeper into the fabric [5,14,30,64]. Therefore, it is natural that shielding and other related parameters—absorption, reflection, and transmission—depend both on the coating deposit and on the structural parameters and finishing of the fabric that is used as a substrate for the coating.

In order to analyze these dependencies, fabrics of different structures were coated with the same conductive paste containing PEDOT:PSS, applying various coating deposits.

For the samples developed using the knife-over-roll coating technique, the coating deposit was varied by controlling the blade height above the fabric and the speed of fabric movement in the line. In the case of screen printing, the amount of deposit was alternated by choosing a different number of passes.

It can be observed (see Supplementary Table S2: Data of samples coated using the knife-over-roll technique; and Table S3: Data of samples coated using the screen-printing technique) that the investigated microwave properties of developed samples are highly dependent on the coating deposit. The electrical properties can be adjusted to a certain degree and thus affect the microwave properties of the coated material by controlling the coating deposit on the fabric.

For each type of fabric, the coating deposit, along with the quantity of the conductive additive, has a significant impact on the shielding properties. Therefore, based on the obtained data, it can be stated that, for particular fabric types, the SE determined is as a function of coating deposit. This dependency is also clearly visible in Figure 4, which shows the variation in SE depending on the coating deposit for each different type of fabric over the whole tested frequency range. It can be seen that, for substrate PC, which was coated via the knife-over-roll technique (Figure 4a,b)—with increasing coating deposits of 7 g/m^2^, 11 g/m^2^, 14 g/m^2^, 17 g/m^2^, and 23 g/m^2^ demonstrating increased |SE| values of 13 dB, 15 dB, 15 dB, 20 dB, and 25 dB, respectively—the coefficient of determination is R^2^ = 0.96 (see Supplementary Figure S2: Dependence of SE on coating deposit for substrate PC). A comparable correlation between the coating deposition and the shielding effectiveness was observed for another type of fabric under this study—substrate code NV (Figure 4c,d). In this case, the same coefficient of determination, i.e., R^2^ = 0.95, was obtained (see Supplementary Figure S3). For other substrates—codes C (Figure 4e,f), P (Figure 4g,h), and AV (Figure 4i,j)—coated by the same technique, we can also observe the same trend: by increasing the coating deposit, the SE increases.

**Figure 5:**
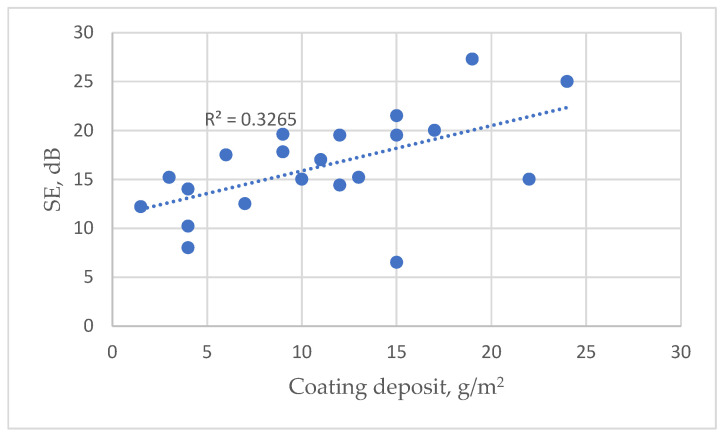
Dependence of SE on coating deposit of all tested fabrics.

However, the experimental data indicate distinctions in the EMR shielding effectiveness among different types of coated fabrics (see Figure 5). It demonstrates that the dependence of the SE on the coating deposit is relatively low across all tested fabrics with different substrates, as indicated by the coefficient of determination (R^2^ = 0.33). However, when considered individually for each type of fabric, this coefficient substantially increases (see Supplementary Figure S2: Dependence of SE on coating deposit for substrate PC; and Figure S3: Dependence of SE on coating deposit for substrate NV). Based on the presented data, it can be stated that SE can be considered a function of coating deposit only for a particular type of fabric—in our case, only for fabrics with the same substrate.

**Figure 6:**
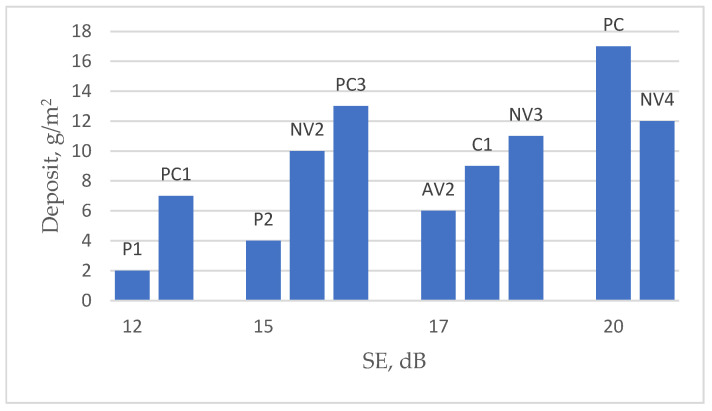
Dependence of SE on coating deposit of fabrics coated via the knife-over-roll technique.

It can be observed that fabrics of different structures, even with the same or very similar SE, have quite significantly different coating deposits (see Figure 6).

The obtained data confirm the statements of other authors [5,14,63] that not only the composition and amount of conductive additive but also the parameters of the fabric structure significantly influence the EMR shielding properties of shielding textiles.

The impact of certain structural parameters of fabrics that are used as substrates for coating is of great importance, as they can strongly influence the permeability properties, which play a crucial role during the coating process.

Such structural parameters of fabric as the density, thickness, mass per unit area, and porosity (number, size, and distribution of pores of woven fabric) determine how conductive paste will penetrate through the fabric.

Naturally, the pretreatment of the fabric and the method of coating also have a significant effect on this process.

Meanwhile, the fiber composition of fabrics mostly influences the bonding between the fibers and the polymer coating [46].

In order to determine the influence of the structural parameters of the substrate on the coating deposit and shielding, the measurement results obtained for fabrics with different structures were further analyzed, taking into account the coating method.

In order to eliminate the influence of the substrates’ surface properties (hydrophobicity or hydrophilicity), samples possessing the substrates with the same aqueous liquid repellency (level 0) and similar moisture management parameters (wetting time and absorption rate; see Table 1) were selected for this analysis—substrates with codes PC, NV, P, and AV. These samples were coated using two techniques—knife-over-roll and screen printing. For coating via screen printing, the substrate with code NV was not used, as it is too thin, and, in this case, the paste penetrates to the other side of the fabric.

Examining the influence of fabric structure, in this study, the following parameters were considered: density, thickness, and mass per unit area. Instead of evaluating the porosity parameters, air permeability was considered, since this parameter is closely related to the size and distribution of pores in the woven fabric.

**Figure 7:**
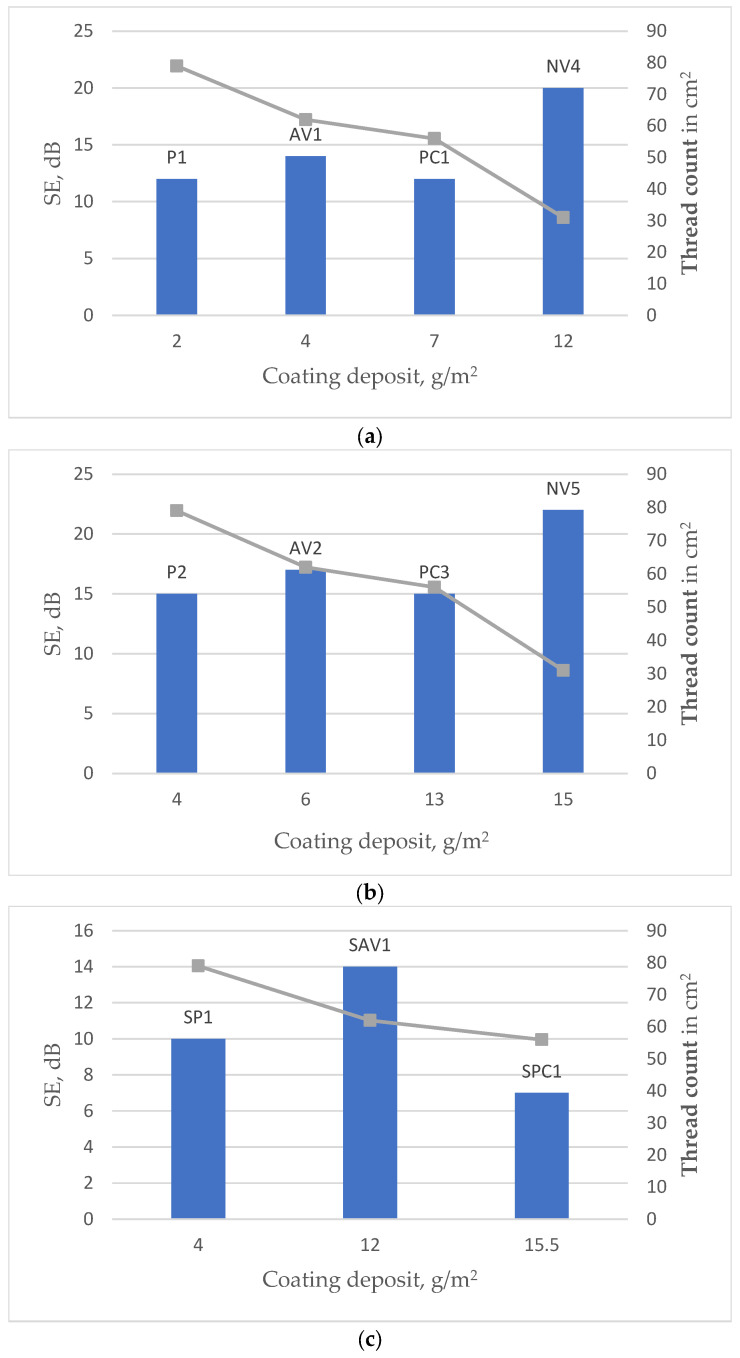

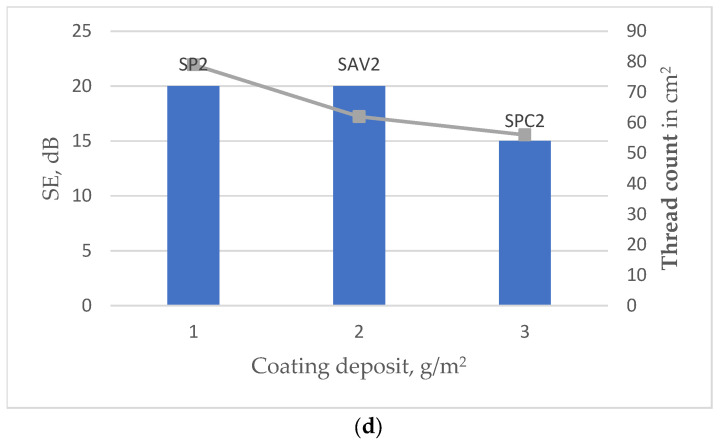
Effectiveness of EMR shielding, coating deposit, and thread count in m^2^ for different types of fabrics: (**a**) fabrics coated via the knife-over-roll method, with the gap between knife and fabric being 0.1 mm; (**b**) fabrics coated via the knife-over-roll method, with the gap between knife and fabric being 0.2 mm; (**c**) fabrics coated via the screen-printing method, with the number of passes being 4; (**d**) fabrics coated via the screen-printing method, with the number of passes being 6.

Figure 7 shows the effectiveness of EMR shielding effectiveness (dominant for each sample over all tested frequency ranges) and coating deposit for different types of fabrics coated via the knife-over-roll method (Figure 7a,b) or via screen printing (Figure 7c,d). The columns in the diagram show the SE value for a specific deposit, while the points located above each column indicate the thread count in cm^2^ of the substrate used. The points are connected by lines to illustrate the decreasing trend in density. The data presented in Figure 7 show that, in all cases, the deposit increases as the density of the substrate fabric decreases. The parameter characterizing the density—the thread count, which is given in cm^2^—for different substrates used is as follows: code P-79, code AV-62, code PC-56, code C-34, code NV-31 (also see Table 1).

It is possible to observe that samples with different substrates, even coated in the same conditions, differ in coating deposit; however, a higher deposit amount does not always determine a higher shielding.

In the case of the knife-over-roll method (see Figure 7a,b), the highest SE and deposit among tested fabrics demonstrate a sample with an NV substrate, which has significantly lower density and much higher porosity than other types of substrates (see Figure 1). This can be explained by the fact that, even when different substrates are coated under the same conditions, the sample with the NV substrate remains more porous (see Supplementary Figure S4: SEM images of fabric surfaces obtained using a knife-over-roll coating method, with a 0.1 mm gap between the knife and the fabric) and allows for more conductive paste to penetrate it. Consequently, this results in a larger surface area coverage on the thread compared to denser substrates, where the coating predominantly forms on the fabric’s surface.

But other tested fabrics (based on substrates P, AV, and PC) behave differently—as the deposit increases, the shielding efficiency does not increase, and, in some cases, it even decreases (Figure 7a,b). A similar trend is observed in the case of screen printing (see Figure 7c,d).

Comparing the fabrics based on different substrates—P, AV, and PC—it can be seen that samples based on the substrate PC (samples PC1, PC3, SPC1, and SPC2), even with a larger amount of coating deposit, reach only a similar SE as (see Figure 7a,b) or a rather lower SE than (see Figure 7c,d) coated samples based on substrates P and AV.

**Figure 8:**
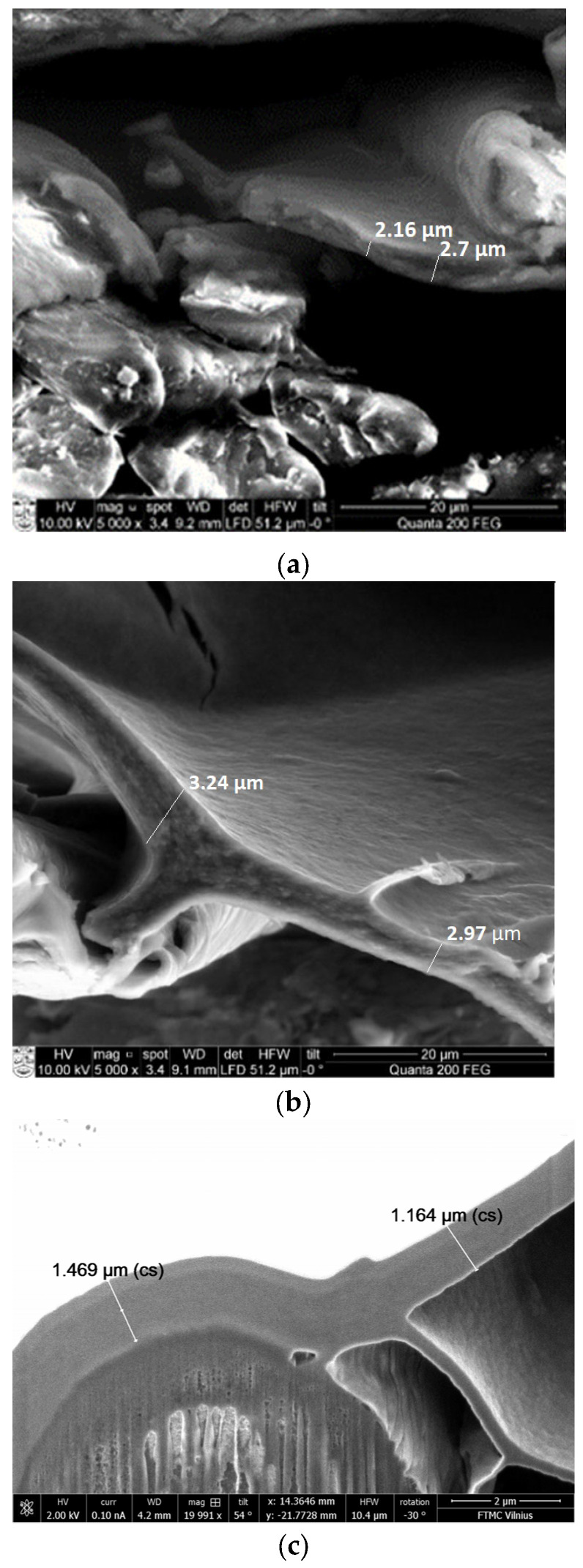
Cross−sectional SEM images of samples coated with knife−over−roll technique: (**a**) sample P2, with coating deposit of 4 g/m^2^ (SE = 15 dB), magnification 5000×; (**b**) sample AV2, with coating deposit of 6 g/m^2^ (SE = 17.5 dB), magnification 5000×; (**c**) sample PC3, with coating deposit of 14 g/m^2^ (SE = 15 dB), magnification 20,000×.

This could be explained by the fact that the PC substrate has a lower density, and, as a result, the paste penetrates deeper into the material. Therefore, as illustrated in the cross-sectional SEM images below (Figure 8), it is evident that the thickness of the coat layer on the top, serving as a shield, is less compared to denser fabrics.

For the samples with the denser substrates—P2 and AV2—the thickness of the coat on the top is almost twice as large as that for sample PC3 (substrate PC), although all three samples are coated under the same conditions (see Figure 8). Thus, the denser the substrate used for coating, the more conductive paste solidifies on the surface, forming a thicker coat on the top.

Therefore, it can be said that, in the case of the PC substrate, more paste penetrates into the deeper layers of the fabric, compared to the P and AV substrates, while the layer on the top remains thinner.

So, it can be stated that relying on fabric structural parameters such as mass per unit area, density, and porosity, a layer of coating paste can just adhere to the fabric surface or penetrate into the fabric, thereby altering the shield thickness and affecting the SE results.

Moreover, based on the received experimental data (see Supplementary Table S2: Data of samples coated using the knife-over-roll technique; Table S3: Data of samples coated using the screen-printing technique), it can be said that, in the case of coated fabrics, in addition to the already listed structural parameters (see Table 1) of the fabric, the shielding properties are also influenced by the technique of coating used.

#### 3.2.3. Influence of Coating Technology on Microwave Properties

When investigating the influence of coating technology used, it was found that, in both cases—screen-printing and knife-over-roll coating—for each type of fabric, an increase in the coating deposition and, consequently, the amount of conductive additive leads to higher SE. However, it was noticed that, to achieve a particular SE value using the knife-over-roll coating technique, the required coating deposit amount is considerably lower as compared with the deposit necessary in the screen-printing case.

**Figure 9:**
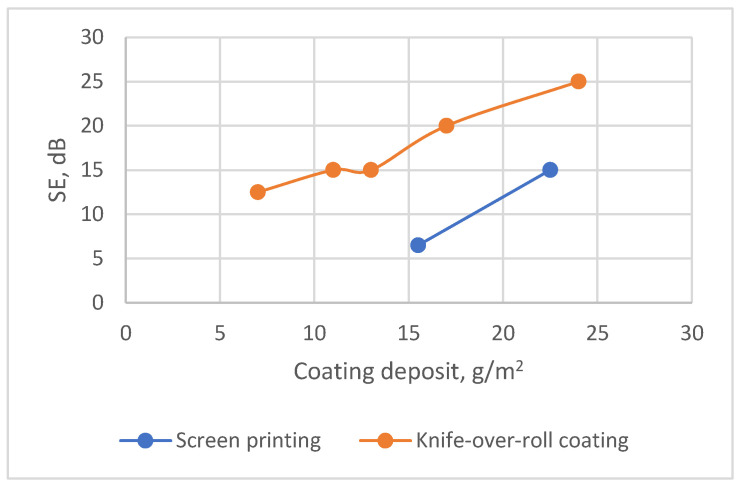
Influence of coating technology used on shielding effectiveness.

For a deeper analysis of the coating technique’s impact, samples of coated fabrics with substrate PC were selected, since most samples in this study were developed namely on this substrate. Figure 9 shows the difference in SE dependence on coating deposit for samples coated on PC substrate using two different coating technologies—screen-printing and knife-over-roll coating. It can be seen from the data presented that the samples covered by screen printing, in order to achieve a certain level of shielding, must have a larger coating deposit than the samples coated by the knife-over-roll technique.

For example, when comparing two samples with a similar SE coated by a different technology, it can be seen that for the knife-over-roll-coated sample (PC3) to reach SE 15 dB, the required deposit needs to be about 14 g/m^2^; meanwhile, for sample SPC2, which was coated by screen printing, this amount rises up to 23 g/m^2^ (see Figure 9).

**Figure 10:**
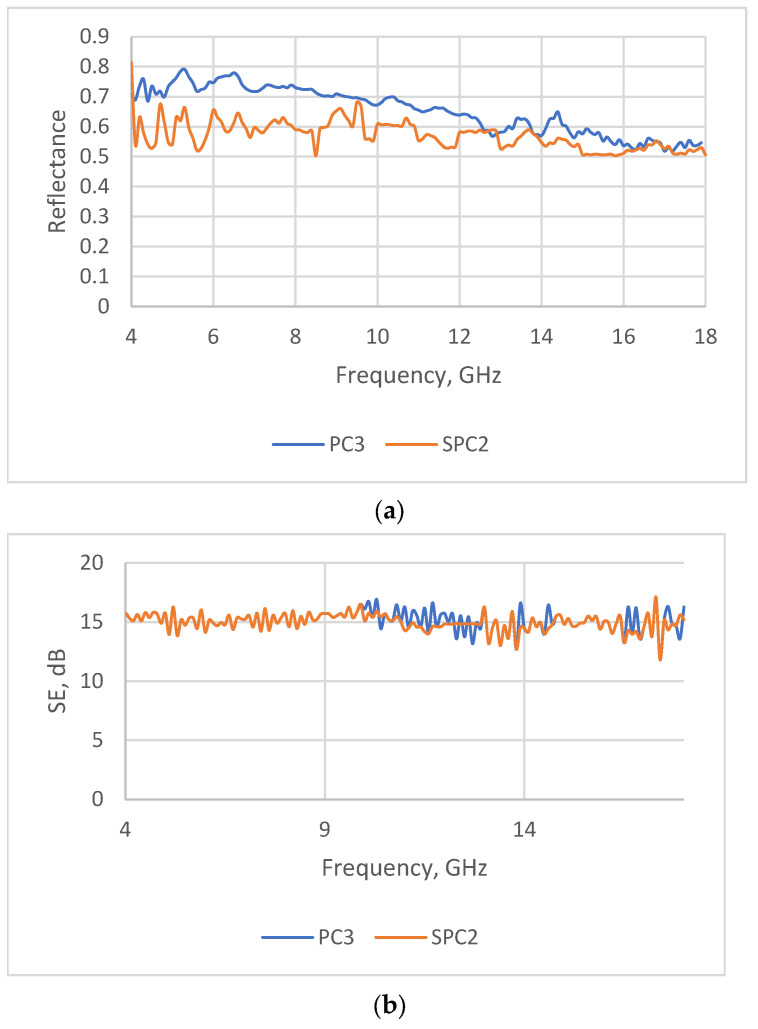
Comparison of microwave properties of samples PC3 and SPC2: (**a**) shielding effectiveness and (**b**) reflectance values.

So, it could be stated that, when using the knife-over-roll method, the coating composition (paste) mostly sticks on the fabric surface, and in the case of screen printing, besides the topcoat, it penetrates into the fabric structure, thus increasing the shield thickness. Consequently, the screen-printed sample (SPC2) shows lower reflectance or has a better absorption ability in the tested range compared with sample PC3 (see Figure 10). These results are in agreement with the EM shielding theory, as absorption ability depends a lot on the shield thickness [5,58].

**Figure 11:**
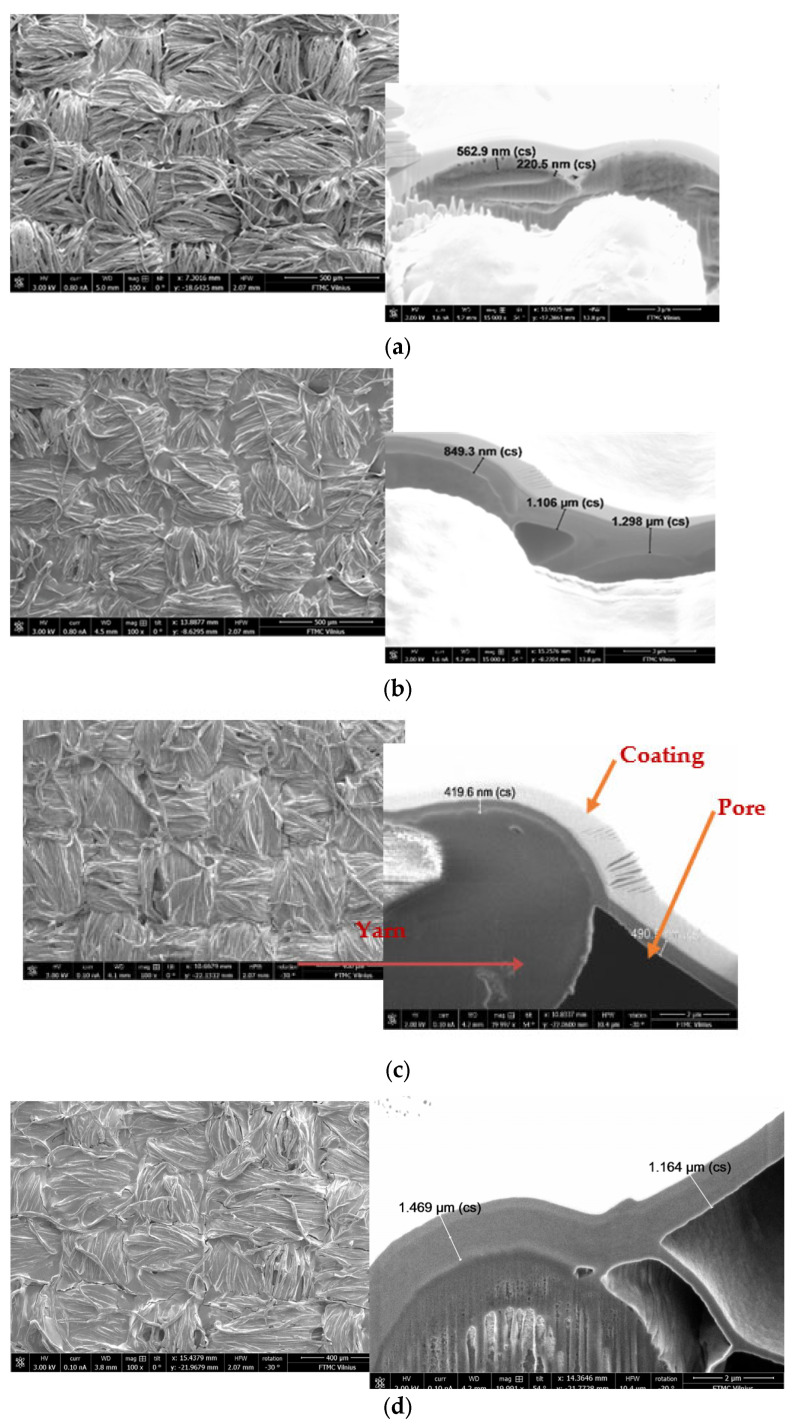
SEM images of PA/cotton fabrics coated with conductive paste: (**a**) sample SPC1, coated by screen printing, coating deposit of 14 g/m^2^; (**b**) sample SPC2, coated by screen printing, coating deposit of 23 g/m^2^; (**c**) sample PC1, coated by knife−over−roll coating, coating deposit of 7 g/m^2^; (**d**) sample PC3, coated by knife−over−roll coating, coating deposit of 14 g/m^2^ (magnification of all SEM images 5000× and 10,000×, respectively).

The scanning electron microscopy analysis of coated samples also confirmed the differences between screen-printed and knife-coated samples (see Figure 11 and Table 4). It is clearly seen that, for example, to obtain a topcoat thickness of about 1 micrometer, the coating deposit for the screen-printed sample needs to be about 23 g/m^2^, while for the knife-coated samples, it needs to be only 14 g/m^2^.

## 4. Wearing Resistance Performance of PEDOT:PSS-Coated Fabrics

Durability, especially resistance to washing and the ability to withstand other impacts that occur during wear, is very important for textile-based shielding materials intended for wearable applications.

However, the main disadvantage of textile and PEDOT:PSS composites is rather low resistance to washing and other wet treatments (wet rubbing or abrasion), due to the solubility of PEDOT:PSS in water.

The fiber composition of the substrate also influences the bonding between the fibers and the polymer coating. Reference [65] identified PEDOT:PSS as a “conductive acid dye” that is capable of tightly binding to protein fibers through electrostatic interactions between the negatively charged sulfonate (-SO3-) ions of the PSS chain and cationic sites of the protein fibers. Since acid dyes are water-soluble anionic dyes, they are used primarily for nitrogenous fibers such as wool, silk, and polyamide, all of which contain basic groups.

To improve the wearing resistance of the PEDOT:PSS-coated fabrics, we applied two methods: the surface modification technique; and atmospheric plasma treatment and chemical modification using a crosslinking agent. The results of the research regarding plasma treatment were presented in our previous articles [46,66]. The plasma treatment proved to be highly effective in enhancing the adhesion strength between the fibers and the conductive composition with PEDOT:PSS. This improvement can be attributed to the incorporation of additional functional groups resulting from plasma reactions, which alter the surface chemistry of the textile substrate through polymer chain decomposition and oxidation [67]. However, the increase in washing resistance after plasma treatment was not significant, mainly because the binding strength of the coat was inadequate to withstand such an impact [46].

A more substantial enhancement in the resistance to the washing of different coated fabrics that had the same substrates as were used in this study was achieved through experiments involving the addition of specific crosslinking agents to a conductive composition containing PEDOT:PSS. Part of the obtained results was included in a patent application [68]. Further findings related to the application of crosslinking agents to improve the washability of textile-based PEDOT:PSS-coated materials are the subject of our upcoming publication.

## 5. Conclusions

Five types of woven fabrics with different structural parameters and various fiber compositions underwent coating with electrically conductive formulation incorporating an intrinsically conductive polymer—PEDOT:PSS. The microwave properties of all samples constructed for this study were investigated within the 2–18 GHz frequency range.

It was determined that, through the regulation of the coating deposit on the fabric, it is feasible to adjust the electrical characteristics, thereby exerting an influence on the reflection and transmission parameters of the coated textile material.

Whereas the electrical conductivity of the fabric is one of the key factors influencing its EMR shielding, an assessment of this property in the textile fabrics with conductive coatings was conducted through the measurement of the inverse dimension—resistance. However, linear resistance, which is obtained for tested coated samples with non-indiscrete surfaces, is not reliable because of the methodology employed, as this method does not eliminate contact resistance within the system and does not allow for the objective prediction of the shielding ability of such fabrics. Thus, the most reliable way to evaluate the shielding effectiveness (SE) of coated fabrics is by physically measuring their transmission.

Transmission measurements showed that the shielding properties of the investigated coated textile fabrics are steady across all examined frequency ranges and are sufficiently good for shielding or radar-absorbing materials. Meanwhile, reflection measurements indicated that both reflection and, as a result, absorption vary with frequency. For all investigated samples, the better absorption ability was achieved within the 12–18 GHz range. It was found that, for fabrics coated with conductive formulation on one side (in our case on the back side), better reflection reduction is obtained when the SE is below |20| dB.

The reflection and transmission measurements revealed that, for tested coated samples, the combined impact of reflection and absorption determines the shielding properties. However, the significance of these two parameters varies among different samples. Thus, the obtained results confirm that not only the composition of the conductive shield but also the parameters of the fabric (used as a substrate for coating) structure (density, thickness, mass per unit area, and air permeability) and their pretreatment before coating affect the EMR shielding, reflection, and absorption properties. In the meantime, the fiber content of textile fabrics mostly influences the bonding between the fibers and the polymer coating.

While investigating the influence of coating technology used, it was found that, in both cases (screen-printing or knife-over-roll coating), increasing the coating deposit, as well as the quantity of conductive additive, leads to a higher SE. However, to achieve a particular SE value using knife-over-roll coating technology, the required coating deposit amount is considerably lower as compared with the deposit necessary in the screen-printing case.

## Figures and Tables

**Table 1:** Characterization of woven fabrics (substrates).

Substrate (Code, Weave, and Fiber Composition)	Mass per Unit Area, g/m^2^	Thread Count in cm^2^	Thickness, mm	Air Permeability, mm/s	Moisture Management		Aqueous Solution Repellency Grade
					Wetting Time, Grade	Absorption Rate, Grade	
PC (plain) 58% Cotton 42% Polyamide	227	56	0.60	25.5	3–4	3–4	0
NV (plain) 50% Nomex 50% Viscose	140	31	0.44	1315	4	3	0
C (plain Cordura) 100% Polyamide	215	34	0.50	70	3	1	2
P (twill) 100% Polyamide	122	79	0.35	62.3	4	3–4	0
AV (ripstop) 55% Aramid 45% Viscose	247	62	0.51	52.7	3	3	0

**Table 2:** Information about the conductive paste applied to the substrates (information presented by supplier).

Composition/information on ingredients (component name)	Benzenesulfonic acid, ethenyl-, homopolymer, compound with 2,3-dihydrothienol[3,4-b]-1,4-dioxin homopolymer
Concentration	1–3% *w*/*w*
Surface resistivity (test print)	700 Ω/sq
Product description (supplied form)	Aqueous dispersion
Dynamic viscosity	3.000 mPa × s (23 °C)
Kinematic viscosity	>40 mm^2^/s (23 °C);
	>20.5 mm^2^/s (40 °C);
Weight ratio (PEDOT:PSS)	1:2.5

**Table 3:** Grading table of moisture management properties.

	Grade	1	2	3	4	5
Index						
Wetting Time, s		≥120 No wetting	20–119 Slow	5–19 Medium	3–5 Fast	<3 Very fast
Absorption Rate, %/s		0–9 Very slow	10–29 Slow	30–49 Medium	50–100 Fast	>100 Very fast

**Table 4:** Comparison of samples with similar SE coated using different technologies.

Sample	Coating Method	Coating Deposit, g/m^2^	Coating. Thickness, µm	Dominant Shielding Effectiveness (SE), dB	Reflectance (R)
PC3	Knife-over-roll coating	14	1.2–1.5	15	0.7–1
SPC2	Screen printing	23	0.85–1.2	15	0.5–0.6

## Data Availability

The data presented in this study are available upon request from the corresponding author.

## Supplementary Materials

The following supporting information can be downloaded at https://www.mdpi.com/article/10.3390/polym15214224/s1, Table S1: Mean value of linear resistance of different fabrics coated with various amounts of conductive paste containing PEDOT:PSS; Table S2: Data of samples coated using the knife-over-roll technique; Table S3: Data of samples coated using the screen-printing technique; Figure S1: Dependence of linear resistance on coating deposit; Figure S2: Dependence of SE on coating deposit for substrate PC; Figure S3: Dependence of SE on coating deposit for substrate NV; Figure S4: SEM images of fabric surfaces obtained using a knife-over-roll coating method, with a 0.1 mm gap between the knife and the fabric.
