# Supplementary material for: The Impact of Structural Variations and Coating Techniques on the Microwave Properties of Woven Fabrics Coated with PEDOT:PSS Composition
Source: Polymers (Basel). 2023 Oct 25;15(21):4224. doi: 10.3390/polym15214224 (PMC10649923; doi:10.3390/polym15214224)
Supplement: Supplementary file 1 [file polymers-15-04224-s001.zip › polymers-2651047-supplementary.pdf]

SUPPLEMENTRAY MATERIAL

Table S1. Mean value of linear resistance of different fabrics coated with various amounts of conductive paste, containing PEDOT:PSS.

Code of sample	Coating deposit, g/m^2	Dominant SE, dB	Linear resistance, Ω/cm
**PC1**	7	12.5	959
**PC2**	11	14.5	218.9
**PC3**	14	15	199.8
**PC4**	17	20	148.3
**PC5**	23	25	117.37
**NV1**	4	7.5	484.4
**NV2**	10	15	371.5
**NV3**	11	17.5	108.6
**NV4**	12	19.5	73.9
**NV5**	15	20.5	43.6
**C2**	19	27	34.68
**C1**	9	18	109.7
**SAV1**	12	14.6	44.7
**SAV2**	15	19	21.4
**SP1**	4	10.3	68.6
**SP2**	9	20	19.9

Table S2. Data of samples coated using the knife-over-roll technique.

Substrate	Code of sample	Coating deposit g/m²	Gap between knife and fabric, mm	Frequency, GHz	Shielding effectiveness SE, dB	Components of shielding effectiveness, %		
						Trans-mission	Reflection	Absorption
PC	PC1	7	0.1	10	12.5	6	70	24
				12	12.4	6	65	29
				18	13	5	50	45
	PC2	11	0.2	10	15	2	95	3
				12	15	2	90	8
				18	15	2	65	33
	PC3	14	0.25	10	15	3	70	27
				12	15.2	3	62	35
				18	15.4	3	58	39
	PC4	17	0.3	10	20	1	90	9
				12	19.8	1	85	14
				18	20.2	1	70	29
	PC5	23	0.5	10	25.2	~ 0	95	5
				12	25	~ 0	90	10
				18	24.8	~ 0	78	22
NV	NV1	4	0.25	10	7.5	20	30	50
				12	8	20	30	50
				18	8.2	20	25	55
	NV2	10	0.5	10	15	3	75	22
				12	15.2	3	72	25
				18	15.4	3	70	27
	NV3	11	0.1	10	17.5	2	80	18
				12	17	2	70	28
				18	17.5	2	65	33
	NV4	12	0.15	10	20	1	80	19
				12	19	1	70	29
				18	20	1	50	49
	NV5	15	0.2	10	21	1	92	7
				12	21.5	1	90	9
				18	22	1	75	24
C	C1	9	0.2	10	17.5	2	85	13
				12	18	2	80	18
				18	17.8	2	65	33
	C2	19	0.5	10	27	~ 0	90	10
				12	27.5	~ 0	85	15
				18	27.5	~ 0	70	30
P	P1	2	0.1	10	12	6	50	44
				12	12.5	6	50	44
				18	12.2	6	35	59
	P2	4	0.2	10	15.2	3	65	32
				12	15	3	65	32
				18	15.3	3	60	37
AV	AV1	4	0.1	10	13.8	4	65	31

				12	14,2	4	55	41
				18	14	4	50	46
	AV2	6	0.2	10	17.5	2	70	28
				12	18	2	65	33
				18	17.2	2	60	38

Table S3. Data of samples coated using the screen-printing technique.

Substrate	Code of sample	No. of passes	Coating deposit, g/m²	Frequency, GHz	Shielding effectiveness SE, dB	Components of shielding effectiveness, %		
						Transmission	Reflection	Absorption
PC	SPC1	4	14	10	6.5	20	50	30
				12	6.5	20	40	40
				18	6.8	20	35	45
	SPC2	6	23	10	15	3	60	37
				12	14.8	3	58	39
				18	15.2	3	50	47
P	SP1	4	4	10	10.3	9	65	26
				12	10.4	9	67	24
				18	10.1	10	64	26
	SP2	6	9	10	20	1	85	14
				12	19.6	1	76	23
				18	19.5	1	89	10
AV	SAV1	4	12	10	14.6	4	68	28
				12	14.3	4	64	32
				18	14.3	4	68	28
	SAV2	6	15	10	19.6	1	79	20
				12	19.5	1	76	23
				18	19.2	1	71	28

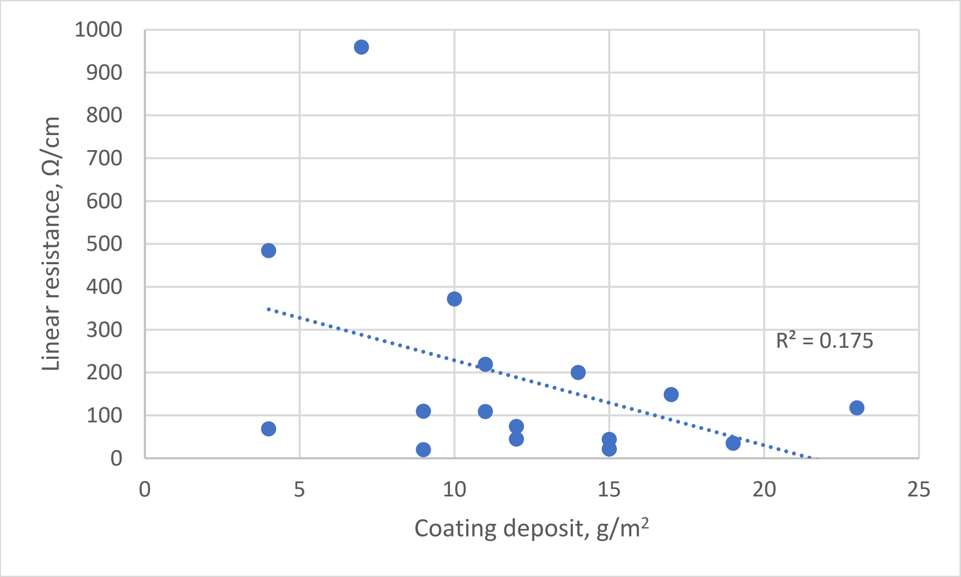

Figure S1. Dependence of linear resistance on coating deposit.

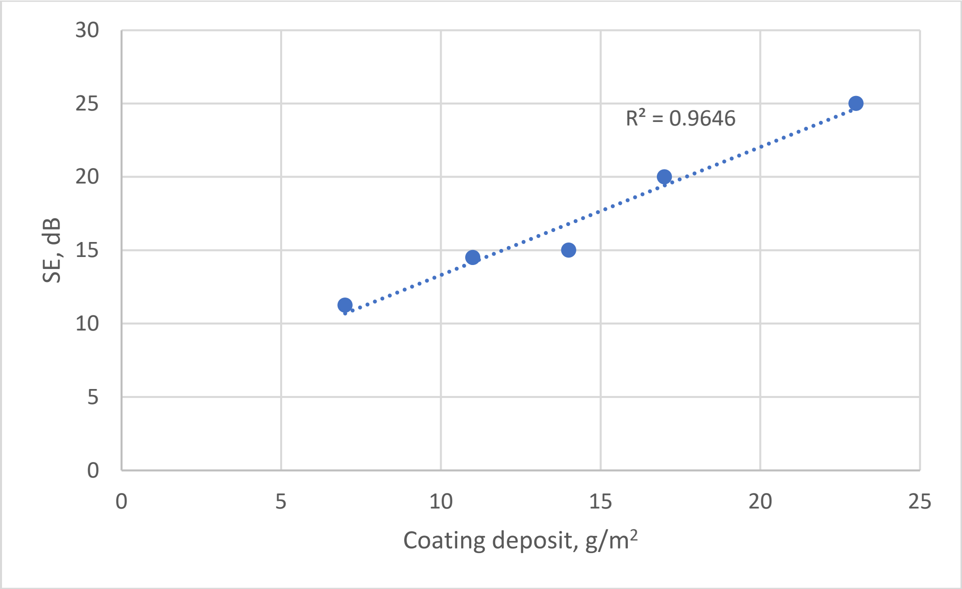

Figure S2. Dependence of SE on coating deposit for substrate PC.

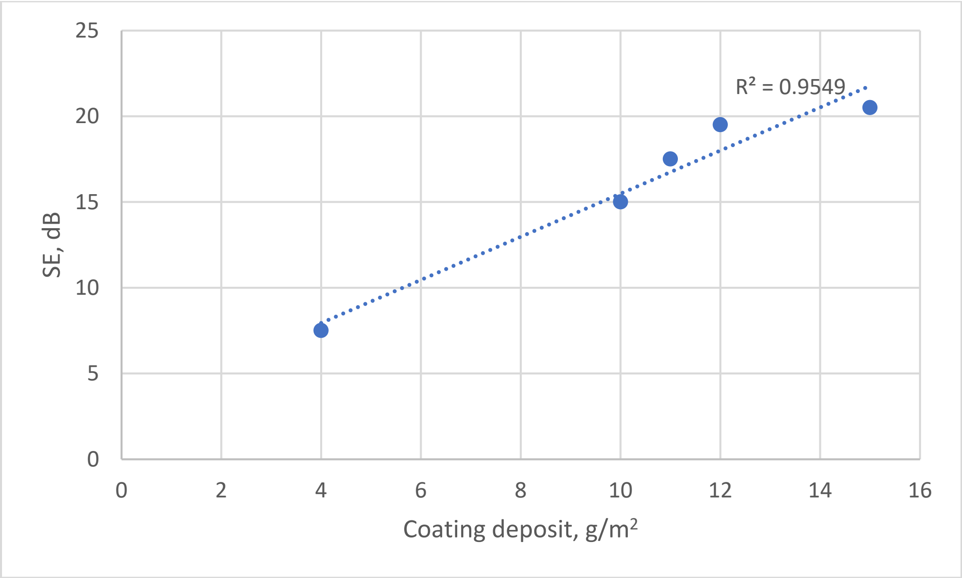

Figure S3. Dependence of SE on coating deposit for substrate NV.

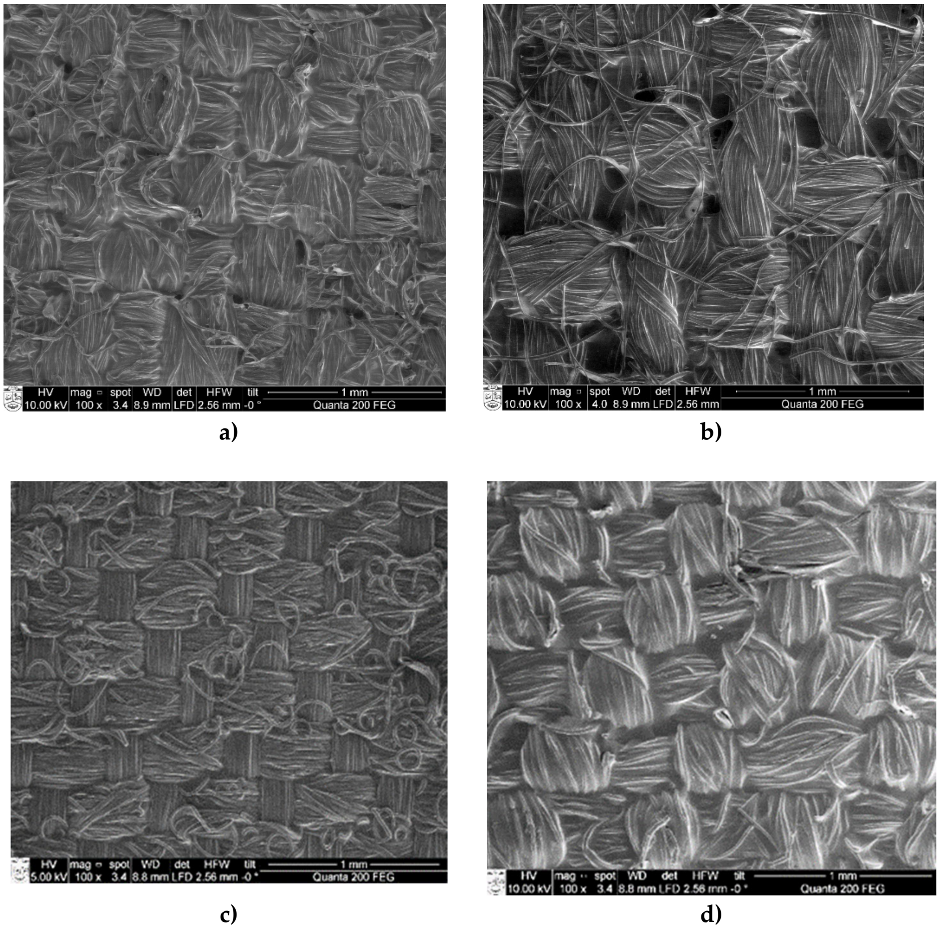

Figure S4. SEM images of fabric surfaces obtained using a knife-over-roll coating method, with a 0.1 mm gap between the knife and the fabric: (**a**) PC1, (**b**) NV3, (**c**) P1, (**d**) AV1.
